# Evaluation of Selected Brain Damage Biomarkers in the Determination of Brain Damage in Dogs With Neurological Distemper

**DOI:** 10.1002/vms3.70894

**Published:** 2026-04-10

**Authors:** Muhammed Mustafa Kapar, Mahmut Ok, Oğuzhan Avcı

**Affiliations:** ^1^ Department of Internal Medicine, Faculty of Veterinary Medicine Selcuk University Konya Turkey; ^2^ Department of Virology, Faculty of Veterinary Medicine Selcuk University Konya Turkey

**Keywords:** brain damage biomarker, dog, neurological distemper

## Abstract

**Background:**

Canine distemper is a disease with high morbidity and mortality in domestic and wild carnivores caused by Canine distemper virus (CDV). Respiratory, gastrointestinal, dermatological, ophthalmic, or neurological disorders occur in dogs with canine distemper. CDV replicates in the central nervous system and causes non‐purulent encephalomyelitis. This causes damage to the brain and spinal cord.

**Objectives:**

The aim of the study was to reveal brain damage caused by the canine distemper virus in dogs using selected brain damage biomarkers and to determine which of these biomarkers are diagnostically significant in detecting brain damage.

**Methods:**

Thirty‐six six dogs with neurological of distemper and 16 control uninfected dogs were included in the study. Dogs with neurological distemper have nasal discharge, myoclonus of the temporal, chest, back and leg muscles, hyperkeratosis of the tip of the nose and footpad (Hard‐pad). The disease was diagnosed by CDV Ag ELISA test and Polymerase chain reaction (PCR) test. A complete blood count was performed on the blood sample with K‐EDTA. Venous blood gas measurement was performed in heparinised blood samples. The concentrations of neuron‐specific enolase (NSE), glial fibrillary acidic protein (GFAP), creatine kinase BB (CK‐BB), adrenomedullin (ADM), activin A (ACTA), immunoglobulin G (IgG) and immunoglobulin M (IgM) were measured in serum using canine‐specific commercial enzyme‐linked immunosorbent assay ELISA kits.

**Results:**

Serum NSE, GFAP, CK‐BB, IgG, and IgM concentrations in dogs with neurological distemper showed statistically significant increases compared to healthy dogs (*p* < 0.001), while no statistically significant differences were detected in ADM and ACTA concentrations (*p* > 0.05).

There was a statistically significant increase in the WBC, LYM, and MON counts of dogs with neurological distemper compared to healthy dogs (*p* < 0.01).

**Conclusions:**

Serum NSE, GFAP, CK‐BB, IgG and IgM concentrations were found to be significantly increased in dogs with neurological distemper. The results of this study indicate that NSE, GFAP, and CK‐BB biomarkers may have diagnostic significance in determining brain damage.

## Graphical abstract

In this study, a significant increase in serum NSE, GFAP, CK‐B, IgM and IgM concentrations was determined in dogs with nervous distemper. It was shown that NSE, GFAP and CK‐B biomarkers have a useful and reliable diagnostic value in determining brain damage in dogs with nervous distemper

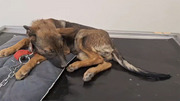

## 1 Introduction

Canine distemper virüs (CDV) is causative agent of a fatal disease in wild and domestic carnivores. CDV is an enveloped, single‐stranded RNA virus belonging to the Paramyxoviridae family and the Morbillivirus genus (Trogu et al. 2021; Chludzinski et al. 2023; McDermott et al. 2023). In dogs, the major route CDV transmission is through aerosols from respiratory exudate containing the virüs, althoug other body excretions and secretions can be infectious CDV is highly contagious, and viral shedding may follow infection for 60–90 days (Deem et al. 2000). Respiratory, gastrointestinal, dermatological, ophthalmic, or neurological disorders occur in dogs with canine distemper (Shell 1990; Beineke et al. 2009). Canine distemper is a highly contagious disease characterised by high morbitidy and mortalite (Trogu et al. 2021). Pathological lesions are mostly observed in the respiratory and gastrointestinal system, lymphoid tissue and central nervous system (Frisk et al. 1999). When CDV replicates in the central nervous system, it causes non‐purulent inflammation in the brain and spinal cord, leading to encephalomyelitis and resulting in chronic neurological disorders. In histopathologically, in the cerebrum, there is multifocal demyelination and necrosis along with mild astrocytosis and lymphoplasmacytic and histiocytic perivascular cuffs (Destri et al. 2020).

In human medicine in recent years, brain damage biomarkers have been used in the diagnosis and prognosis of various central nervous system (CNS) disorders (Serrano et al. 2002; Tomita et al. 2020). In neonates with asphyxia‐induced hypoxic brain injury, significant alterations have been observed in concentrations of glial fibrillary acidic protein (GFAP), S100 calcium‐binding protein B (S100B), neuron‐specific enolase (NSE), ubiquitin carboxyl‐terminal hydrolase L1 (UCHL1), Activin A, adrenomedullin (ADM), and creatine kinase‐BB (CK‐BB) (Douglas‐Escobar et al. 2013).

In perinatal calves with hypoxic–ischaemic encephalopathy, increased levels of UCHL1 and S100B, and decreased levels of ADM, Activin A, NSE, and CK‐BB have been reported (Ok et al. 2022). Similarly, in hypoglycaemic calves, significant increases in S100B, GFAP, UCHL1, and CK‐BB, along with a decrease in Activin A, have been documented (Ider et al. 2023).

ADM, a vasodilatory peptide with hypotensive effects, plays a role in regulating cerebral blood flow during perinatal hypoxia, and elevated blood levels have been associated with neonatal neurological disorders (Di Iorio et al. 2004). Activin A has been reported to exhibit 100% sensitivity and specificity for diagnosing neonatal hypoxic–ischaemic encephalopathy (Florio et al. 2006). NSE levels have also been shown to rise significantly in neonates with asphyxia (Lv et al. 2015). GFAP concentrations significantly increase in perinatal brain injury and may be useful in assessing the extent of damage (Florio et al. 2010). CK‐BB, highly concentrated in brain tissue, increases in peripheral blood following brain injury and is often used alongside S100B as a biomarker of neonatal brain damage (Lyons and Kettenmann 1998; Nagdyman et al. 2001).

In dogs with neurological distemper, IgG levels were found to be significantly increased in both serum and cerobrospinal fluid (Jhonson et al. 1988). Many researchers (Waner et al. 2003; McRee et al. 2014, Sathe and Cusick 2023) have determined IgM and IgG seropositivity in dogs with distemper. Although the presence of distemper‐specific IgM in dogs indicates an acute infection (Waner et al. 2003), it has been demonstrated that IgM remains seropositive in dogs with distemper for between 5 weeks and three months (McRee et al. 2014).

Brain‐specific damage biomarkers have been determined to have diagnostic and prognostic significance in detecting brain damage in disorders affecting the brains of humans and calves. This study was hypothesised based on the idea of revealing brain damage that may occur in dogs with neurological distemper using brain‐specific damage biomarkers. The aim of the study was to reveal brain damage caused by the canine distemper virus in dogs using selected brain damage biomarkers and to determine which of these biomarkers are diagnostically significant in detecting brain damage.

## 2 Material and Methods

### 2.1 Animals

This study was conducted on 36 dogs with neurological distemper and 16 control uninfected dogs. The dogs included in the study were of different sexed and aged between 3 and 12 months. The study was conducted with the approval of the Selcuk University Veterinary Faculty Experimental Animal Production and Research Center Ethics Committee (SUVDAFEK) with decision number 2021/118.

### 2.2 Selection of Dogs With Neurological Distemper

Thirty‐six dogs exhibiting neurological disorders such as myoclonus in the temporal, chest, back, and leg muscles, and hyperkeratosis on the nose tip and footpad (hard pad) were included in the study (Table 1). It was determined that the dogs included in the study had been exhibiting neurological symptoms for 4–6 weeks. The diagnosis of the disease was based on clinical findings, haemogram findings (leukogram), canine distemper virus antigen (CDV Ag) test positivity, and PCR positivity. The canine distemper virus antigen (CDV Ag) test was performed using nasal swabs and conjunctival swabs, while PCR analysis was performed using whole blood and nasal swabs.

**TABLE 1:** Breed, age and sex of dogs with neurological distemper.

Case no	Breed	Age	Sex
**1**	Mixed breed dog	9 months	Male
**2**	Mixed breed dog	5 months	Male
**3**	Kangal Shepherd dog	5 months	Female
**4**	Kangal Shepherd dog	2 months	Female
**5**	Mixed breed dog	7 months	Male
**6**	Golden Retriever	6 months	Female
**7**	Kangal Shepherd dog	6 months	Male
**8**	Mixed breed dog	3 months	Male
**9**	Mixed breed dog	6 months	Female
**10**	Kangal Shepherd dog	6 months	Female
**11**	Kangal Shepherd dog	12 months	Male
**12**	Kangal Shepherd dog	11 months	Female
**13**	Mixed breed dog	6 months	Male
**14**	Kangal Shepherd dog	4 months	Female
**15**	Kangal Shepherd dog	3 months	Female
**16**	Mixed breed dog	4 months	Male
**17**	Mixed breed dog	3 months	Male
**18**	Kangal Shepherd dog	12 months	Female
**19**	Husky dog	6 months	Female
**20**	German Shepherd dog	5 months	Female
**21**	Mixed breed dog	4 months	Male
**22**	Kangal Shepherd dog	4 months	Female
**23**	Kangal Shepherd dog	5 months	Male
**24**	Labrador dog	3 months	Female
**25**	Husky dog	10 months	Male
**26**	Labrador dog	8 months	Female
**27**	Mixed breed dog	3 months	Male
**28**	Rotwieller dog	7 months	Male
**29**	Akbash dog	7 months	Female
**30**	Kangal Shepherd dog	5 months	Male
**31**	Kangal Shepherd dog	12 months	Male
**32**	Kangal Shepherd dog	4 months	Female
**33**	Mixed breed dog	5 months	Male
**34**	Mixed breed dog	4 months	Female
**35**	Mixed breed dog	6 months	Male
**36**	Mixed breed dog	3 months	Female

### 2.3 Selection Healthy Dogs

Sixteen healthy dogs were admitted to the Veterinary Hospital of the Faculty of Veterinary Medicine, Selcuk University for vaccination, preventive antiparasitic administration and general control were enrolled. Dogs without neurological clinical findings, with normal venous blood count and venous blood gas results, and negative CDV Ag ELISA test results were considered healthy and included in the study.

### 2.4 Collection of Blood Samples

Blood samples with and without anticoagulant were collected once from the saphenous vein or cephalic vein of all dogs. A K_3_‐EDTA tube was used for haemogram measurements, an anti‐coagulant‐free tube for serum, and heparinised injectors for blood gas measurements. Haemogram from K‐EDTA blood sample, blood gas from heparinised blood sample and brain injury biomarkers from serum samples were measured. Haemogram and blood gas analyses were performed within 5–10 min. Blood samples collected for biomarker analyses were held at room temperature for 15 min, then centrifuged at 20 × *g* for 10 min, and sera were collected and stored at –80°C until for biomarker analysis.

### 2.5 Polymerase Chain Reaction (PCR) Collection of Samples

Blood sample and nasal swab samples were collected once from 36 dogs suspected of having neurological distemper. Blood samples were kept at room temperature for 1 h and centrifuged at 3000 (VWR, CompactStar CS 4, England) for 10 min. Nasal swabs were collected using commercially available sterile swabs. After mixing the swab tubes by vortexing (Isolab, Germany), the buffer liquid was transferred to 1.5 mL DNAse RNAse free tubes and stored at –20°C.

### 2.6 Blood Analysis

Venous blood pH, partial pressure of carbon dioxide (pCO2), partial pressure of oxygen (pO2), oxygen saturation (SO2), potassium (K), sodium (Na), ionised calcium (iCa), chlorine (Cl), glucose (Glu), lactate (Lac), base deficit (BE) and bicarbonate (HCO3) were measured using an automated blood gas analyser (ABL 90 Flex, Radiometer, Brea, CA, USA). Total leukocyte (WBC), granulocyte (GRA), monocyte (MON), lymphocyte (LYM), total erythrocyte (RBC), haemoglobin (Hb), haemocrit (Hct) and platelet (PLT) parameters were measured using the MS4e device (CFE 279, Haematology Analyser, Melet Schlosing Laboratories, France) in K3EDTA venous blood samples collected from all dogs.

### 2.7 Brain Damage Biomarker Measurement

Serum NSE, GFAP, ADM, ACTA, CK‐B, IgG and IgM concentrations were measured using canine‐specific ELISA commercial test kits (ELK Biotechnology, Shanghai, China) according to the manufacturer's instructions using an absorbance microplate reader (ELx800 Absorbance Micraplate Microplate Reader, USA). Canine‐specific NSE commercial ELISA kit (Cat No.: ELK9192, Lot: 20334267250), canine‐specific GFAP commercial ELISA kit (Cat No.: ELK9719, Lot: 20334270267), canine‐specific ADM commercial ELISA kit (Cat No.: ELK5647, Lot: 20334260317), canine‐specific CK‐B commercial ELISA kit (Cat No: ELK9543, Lot: 20324331407), canine‐specific ACTA commercial ELISA kit (Cat No: ELK9720, Lot: 20334270270), canine‐specific IgG commercial ELISA kit (Cat No: ELK1302, Lot: 20334247726) and canine‐specific IgM commercial ELISA kits (Cat No: ELK9721, Lot: 20334270271) were used. Intra‐assay coefficient of variation (CV), inter‐assay CV and minimum detectable concentrations (MDC) for biomarkers were ≤8%, ≤10% and >0.63 ng/mL for NSE, ≤8%, ≤12% and >0.16 ng/mL for GFAP, ≤8%, ≤12% and >15.63 pg/mL for ADM, <8%, <10% and >15.63 pg/mL for ACTA, <8%, <10% and >0.79 ng/mL for CK‐B, <8%, <10% and >1.57 µg/mL for IgG and <8%, <10% and >0.16 µg/mL for IgM, respectively.

### 2.8 Canin Distemper Virus Ag ELİSA Test Application

The CDV Ag ELISA test (Vettek Medical, Istanbul) was performed on nasal swabs taken from healthy dogs and dogs suspected of having neurological distemper, according to the manufacturer's instructions. The nasal swabs were placed in the test solution and mixed. Three drops of this solution were applied to the test card. The appearance of two lines on the test card was considered positive, while the appearance of a single line was considered negative. Dogs that tested positive on the CDV Ag ELISA test were considered distemper‐infected and included in the study.

### 2.9 Polymerase Chain Reaction Analysis Procedure

All samples stored at –20°C were thawed. The thawed nasal samples were centrifuged at 3000 rpm for 10 min at +4°C (VWR, CompactStar CS 4, England). After RNA centrifugation, supernatants and other leukocyte samples were extracted using a commercially available kit (QIAampViral RNA Mini Kit, Cat No: 52906, USA) according to the manufacturer's instructions. Extraction products (viral RNA) were stored at –20°C until use.

For the detection of Canine Distemper Virus (CDV) by One‐Step RT‐PCR, the One‐Step RT‐PCR Master Mix Kit (abm MegaFiTM, Cat No: G597, Canada) was used. 50 µL reaction mix: 25 µL 2× buffer, 10 ppm forward primer (Fp) 2 µL and reverse primer (Rp) 2 µL, enzyme (reverse transcriptase) 4 µL, Dnase, Rnase free water 12 µL were prepared and 5 µL RNA was added to this mix and the total mix was made up to 50 µL. At the end of the mixing, the samples were placed in a thermal cycler (Biorad, USA) and the RT‐PCR reaction was performed with an RT step at 60°C for 15 min, enzyme activation temperature at 98°C for 30 s, followed by 10 s at 98°C, 25 s at 54°C, 40 s at 72°C and 32 cycles and a final extension at 72°C for 2 min. For this purpose, the primers were designed specifically for the nucleocapsid protein of the virus and the PCR primer pairs reported in this study were used in accordance with the method of Shin et al. (2004).

The oligonucleotide sequences used in the reaction were F: 5'‐TCTGAGGCAGATGAGTTCTT‐3'; R: 5'‐CCATAGCATAACTCCAGAGAGC‐3'.

At the end of the reaction, the PCR products were transferred to an agarose gel (Vivantis, Malaysia) prepared with 2% 1× TAE (Tris‐Acetic Acid‐EDTA) buffer containing 0.01% gel red (Invitrogen SYBR Safe DNA Gel Stain) and electrophoresed (Serva, Blue Marine 100, Germany) at 100 V current (Serva Blue Power Plus, Germany) for 70 min. At the end of the process, the products were analysed in a gel imaging device using a UV transilluminator and gel documentation system and samples showing a band in the 640 bp region were considered positive for the presence of CDV.

### 2.10 Treatment Protocol

Supportive treatment was administered to dogs with neurological distemper. This consisted of fluid and electrolyte administration, anti‐inflammatories, immunomodulators, antibacterials and anticonvulsants. Sick dogs were given fluid therapy. Ceftriaxone (Cefaday, Biofarma, Istanbul) was administered intramuscularly at a dose of 25 mg/kg every 12 h for 5 days to prevent secondary infections. Analgesics and anticonvulsants were used to manage inflammation and seizures. Dexamethasone (Devamed, Topkim, Istanbul) at a dose of 0.25 mg/kg was administered orally for 3 days, and pregabalin (Lyrica, Pfizer, Istanbul) at a dose of 20 mg/kg was administered as an anticonvulsant for 2–4 weeks. Levamisole (Levaject, AGRAR, Istanbul) was administered subcutaneously at a dose of 2.5 mg/kg three times at 3‐day intervals as an immunomodulator. As supportive treatment, B‐complex vitamins were given to restore appetite and vitamins A, C and E against oxidative product damage due to inflammation in the CNS.

### 2.11 Statistical Methods

In analysing the data in this study, SPSS 25 (IBM Corp. Released 2017. IBM SPSS Statistics for Windows, Version 25.0 Armonk, NY: IBM Corp.) was used. The one‐sample Kolmogorov–Smirnov test was used to assess the assumptions of normal distribution (parametric or non‐parametric) of the data. As the study data showed a parametric distribution, they were presented as mean ± SE (standard error). The independent *t*‐test was used to compare between groups. The Pearson's correlation test was used to determine the correlation between variables. The significance level of the tests was accepted as *p* < 0.05.

## 3 Results

### 3.1 Clinical Findings

Conjunctivitis (36 cases), nasal and lacrimal discharge (36 cases), hyperkeratosis of the nasal tip and base pad (hard pad) in some cases (18 cases), myoclonus in the temporal muscles (36 cases), myoclonus in the chest (24 cases), myoclonus in the back and hind leg muscles (22 cases), and epileptic seizures in some cases (6 cases) were observed in dogs with neurological distemper. While 5 dogs responded to the treatment, 31 dogs did not respond to the treatment and died. Deaths occurred between 3 weeks and 6 weeks. The 5 surviving dogs were followed up for 1 year and no signs of distemper were observed. It was determined that 36 dogs infected with neurological distemper had not been vaccinated against distemper.

### 3.2 Necropsy Findings

Necropsies of three dead dogs were performed and histopathologic examination confirmed that CDV caused brain damage. The necropsy revealed non‐suppurative encephalitis. Lesions were observed in the cerebral cortex, cerebellum, medulla oblongata, and white matter near the fourth ventricle.

### 3.3 Histopathologic Findings

Histopathological examination revealed myelin vacuolisation, spongiosis, oligodendrocyte degeneration, multifocal demyelination, perivascular lymphocytic cell infiltration, microgliosis, neuronal necrosis, and apoptosis. In addition, distemper‐specific inclusion bodies were detected in biopsies taken from the conjunctiva of 15 dogs.

### 3.4 Blood Gas Findings

There was no statistical difference (*p* > 0.05) in blood pH, pCO_2_, pO_2_, sO_2_, K, Na, BE, HCO_3_, glucose, lactate, K and Na levels of dogs with neurological distemper compared to control uninfected dogs (Table 2).

**TABLE 2:** Mean values and significance (mean ± SE) of venous blood gases parameters in control uninfected dogs and dogs with neurological distemper.

Parameters	Control uninfected dogs (*n*: 16)	Dogs with neurological distemper (*n*: 36)	*p* value
pH	7.38 ± 0.008	7.36 ± 0.012	*p* = 0.274
pCO_2_ (mmHg)	40.37 ± 2.3	37.41 ± 0.90	*p* = 0.251
pO_2_ (mmHg)	36.91 ± 2.2	36.86 ± 1.4	*p* = 0.985
S0_2_ (%)	60.6 ± 3.7	57.9 ± 2.7	*p* = 0.550
K (mmol/L)	4.01 ± 0.06	4.02 ± 0.08	*p* = 0.932
Na (mmol/L)	148.73 ± 1.6	150.33 ± 2.4	*p* = 0.239
Glucose (mg/dL)	86.1 ± 3.2	91.2 ± 3.1	*p* = 0.258
Lactate (mmol/L)	1.84 ± 0.09	1.64 ± 0.12	*p* = 0.211
BE (mmol/L)	−2.19 ± 0.78	−3.26 ± 0.64	*p* = 0.299
HCO_3_ (mmol/L)	21.83 ± 0.59	21.34 ± 0.46	*p* = 0.512

*Note*: Significance level were accepted as *p* < 0.05.

Abbreviations: BE, base deficit; pCO_2_, partial carbon dioxide pressure; pH, negative logarithm of hydrogen ion concentration; pO_2_, partial oxygen pressure; SO_2_, oxygen saturation.

### 3.5 Haemogram Findings

There was a statistically significant increase (*p* ˂ 0.01) in WBC, LYM and MON counts, and a decrease (*p* ˂ 0.01) in RBC, MCV, MCHC and Hb values of dogs with neurological distemper compared with control uninfected dogs. However, no statistically significant difference was observed in blood GRA, Hct, and PLT values (*p* > 0.05) (Table 3).

**TABLE 3:** Haemogram parameters means and significance (mean ± SE) of control uninfected dogs and dogs with neurological distemper.

Parameters	Control uninfected dogs (*n*: 16)	Dogs with neurological distemper (*n*: 36)	*p* value	Reference ranges
WBC (m/mm^3^)	12.25 ± 1.0	19.71 ± 1.3	***p* < 0.001**	5.00–17.5
LYM (m/mm^3^)	1.92 ± 0.21	5.82 ± 0.40	***p* < 0.001**	0.20–4.9
MON (m/mm^3^)	0.66 ± 0.08	2.88 ± 0.22	***p* < 0.001**	0.10–1.10
GRA (m/mm^3^)	9.66 ± 0.89	11.00 ± 1.1	*p* = 0.350	2.00–13.20
RBC (m/mm^3^)	7.01 ± 0.25	6.05 ± 0.19	*p* = 0.03	4.00–9.00
MCV (fl)	69.15 ± 1.1	63.46 ± 1.2	***p* < 0.001**	35.50–55.00
Hct (%)	47.10 ± 1.6	43.46 ± 1.3	*p* = 0.091	24.00–45.00
MCHC (g/dL)	32.68 ± 0.57	29.78 ± 0.42	***p* < 0.001**	28.00–40.00
Hb (g/dL)	15.80 ± 0.59	12.85 ± 0.36	***p* < 0.001**	9.50–15.00
PLT (m/mm^3^)	327.00 ± 33.00	311.00 ± 22.00	*p* = 0.699	120.00–500.00

*Note*: Significance level was accepted as *p* < 0.05.

Abbreviations: Hb, haemoglobin; Hct, haematocrit; LYM, lymphocytes; MCHC, mean erythrocyte haemoglobin concentration; MCV, mean erythrocyte volume; MON, monocytes; PLT, platelets; RBC, erythrocytes; WBC, total leukocytes.

### 3.6 Biomarker Findings

There was a statistically significant increase (*p* ˂ 0.01) in serum NSE, GFAP, CK‐B, IgG and IgM concentrations in dogs with neurological distemper compared to control uninfected dogs (Table 4), whereas no difference was found in serum ADM and ACTA concentrations

**TABLE 4:** Means and significance (mean ± SE) of serum biomarker parameters of control uninfected dogs and dogs with neurological distemper.

Parameters	Control uninfected dogs (*n*: 16)	Dogs with neurological distemper (*n*: 36)	*p* value
NSE (ng/mL)	34.01 ± 3.78	66.50 ± 3.77	*p* < 0.000
GFAP (ng/mL)	3.43 ± 1.04	11.87 ± 1.53	*p* < 0.000
CK‐BB (ng/mL)	22.31 ± 2.83	40.63 ± 3.97	*p* < 0.000
ADM (pg/mL)	473.59 ± 34.26	450.27 ± 49.22	*p* = 0.699
ACTA (pg/mL)	306.45 ± 35.13	283.74 ± 39.62	*p* = 0.670
IgG (µg/mL)	23.82 ± 3.49	65.30 ± 9.43	*p* < 0.000
IgM (µg/mL)	3.70 ± 0.66	17.73 ± 1.70	*p* < 0.000

*Note*: Significance level was accepted as *p* < 0.05.

Abbreviations: ACTA, activin A; ADM, adrenomedullin; CK‐BB, creatine kinase‐BB; GFAP, glial fibrillary acid protein; IgG, immunoglobulin G; IgM, immunoglobulin M; NSE, neuron‐specific enolase.

### 3.7 PCR and CDV Ag Findings

**FIGURE 1:**
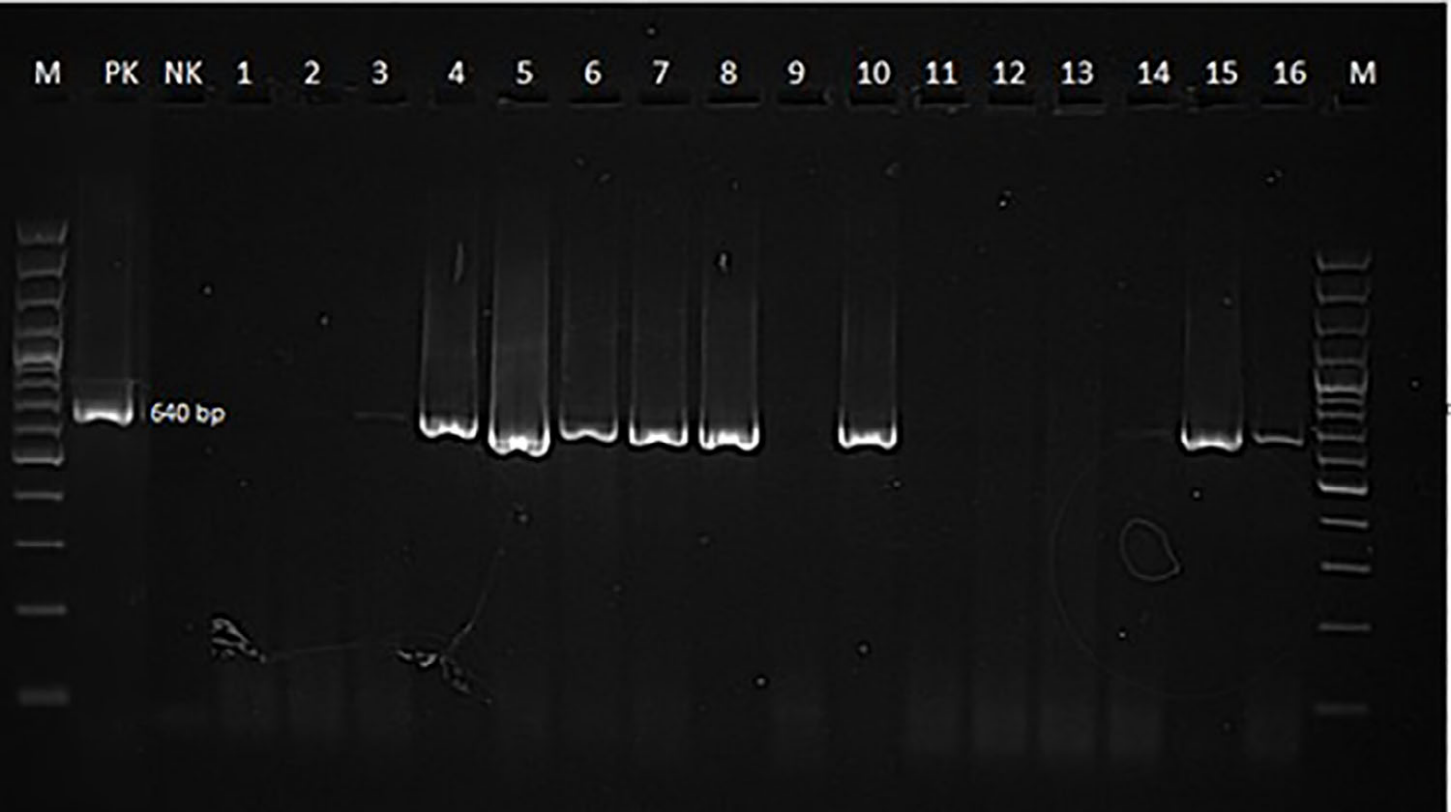
PCR canine distemper virus positivity in a nasal swab from a dog with neurological distemper.

**FIGURE 2:**
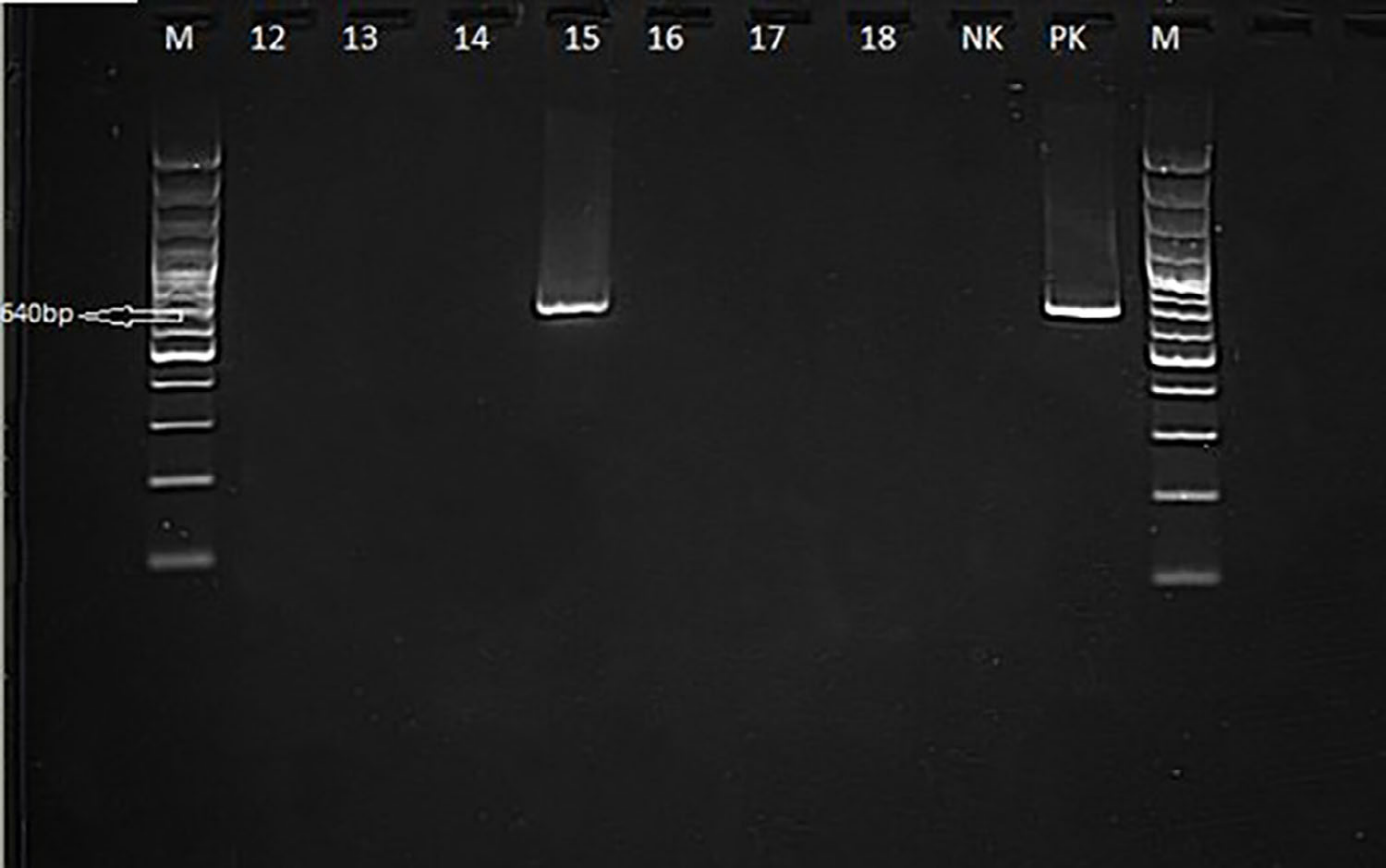
PCR canine distemper virus positivity in whole blood (leukocyte) from a dog with neurological distemper.

Thirty‐six dogs with neurological distemper were tested for CDV Ag in nasal swabs and all cases were positive. PCR was performed on whole blood and nasal swabs from these dogs. PCR analysis detected CDV antigen positivity in nasal swabs (Figure 1) from 31 of 36 dogs and in leukocytes (Figure 2) from 5 dogs.

## 4 Discussion

In this study, changes in the concentrations of the brain injury biomarkers NSE, GFAP, ADM, ACTA, IgM, and IgG, as well as CK‐BB enzyme activity, were evaluated in serum samples from dogs with neurological distemper. The results of the study revealed that brain damage developed in dogs with neurological distemper, as demonstrated by brain damage biomarkers. Significant increases were observed in serum NSE and GFAP concentrations and CK‐BB enzyme activity. Since no studies on this subject have been found in dogs, the discussion is based on human and calf references.

In dogs with distemper, conjunctivitis, nasal and lacrimal discharge, vomiting, diarrhoea, hyperkeratosis at the tip of the nose, and hyperkeratosis on the footpads (hard pads) are observed, neurological distemper in dogs is characterised by partial or complete tetraparesis, cerebellar and vestibular signs, myoclonus, visual impairment, seizures, and dementia (Shin et al. 2004; Amude et al. 2006; Gulersoy et al. 2020; Gulersoy et al. 2022a). In the present study, in accordance with the reports of many researchers (Shin et al. 2004; Amude et al. 2006; Gulersoy et al. 2020; Gulersoy et al. 2022b), conjunctivitis, nasal and lacrimal discharge, hyperkeratosis (Hard‐pad) on the nasal tip and base pad in some cases, myoclonus in the temporal and front leg muscles, chewing‐gum movements in the mouth, myoclonus in the muscles of the chest, back and hind legs in some cases were determined. Epileptic seizures were also detected in some cases.

The most common haematological findings in dogs with distemper are reported to be anaemia and lymphopenia (Tudury et al. 1997). A significant decrease in MCH and MCHC values reflecting microcytic hypochromic anaemia has been reported in dogs infected with CDV (Headly andSukura 2009). Saaed and Al‐Obaidi (2021) reported decreased RBC, Hb, PCV, PLT, and lymphocyte counts in dogs with distemper. It has been reported that the persistence of CDV in the bone marrow may cause erythrocyte hypoplasia and bone marrow suppression, which may affect haematopoietic progenitor cells and lead to a decrease in the production of these cells (Bohn 2013; Carter 2018). In the present study, a statistically significant decrease (*p* ˂ 0.01) in RBC, MCV, MCHC and Hb values was determined in dogs with neurological distemper compared to control uninfected dogs (Table 3). However, since RBC, MCV, MCHC and Hb values were within the normal reference range, they were not considered as anaemia. The most significant change observed in the leukocyte panel during the acute phase of canine distemper is a decrease in leukopenia and lymphocyte count (Tudury et al. 1997; Ezeibe and Udegbunom 2008; Saaed and Al‐Obaidi 2021; Yama et al. 2020). However, it has been reported that there is no change in WBC, LYM, MON or GRA counts in dogs with neurological distemper (Gulersoy et al. 2022a; Gulersoy et al. 2022b). Degirmencay (2023) found significant decreases in WBC, neutrophil and MON counts in acute form distemper. In the present study, statistically significant increases (*p* < 0.01) in the WBC, LYM and MON counts of dogs with neurological distemper were observed compared to control uninfected dogs (Table 3). The results of this study did not support the findings of many researchers (Tudury et al. 1997; Ezeibe and Udegbunom 2008; Saeed and Al‐Obaidi 2021; Degirmencay 2023), because these researchers have reported leukocyte findings in dogs with acute distemper. In acute cases, leukopenia is detected as a result of lymphopenia. The probable reason for the increase in WBC, LYM, and MON counts in dogs with neurological distemper may be related to the cellular response to antigens in the subacute or chronic stages of viral infections, which is given always through monocytes and lymphocytes (Nelson and Couto 2019).

In recent years, brain damage biomarkers have been evaluated in human medicine for the diagnosis and prognosis prediction of various central nervous system disorders (Serrano et al. 2002; Tomita et al. 2020). Brain damage in calves with hypoxic–ischaemic encephalopathy (Ok et al. 2022) and hypoglycaemia (Ider et al. 2023) has been demonstrated using brain damage biomarkers. In this study, the damage caused by the virus in the brains of dogs with neurological distemper was revealed using brain damage biomarkers. NSE, GFAP, IgG, and IgM concentrations, as well as CK‐BB enzyme activity, were found to be significantly increased in dogs with neurological distemper.

Neuron‐specific enolase is a 78‐dalton isoenzyme found in neurons and neuroendocrine tissues. The increase in NSE levels released by astrocytes, oligodendrocytes and neurons is recognised as an indicator of brain damage (Phalman et al. 1984; Marci et al. 2004). Serum and cerebrospinal fluid NSE levels have been found to increase in brain and blood‐brain barrier disorders (Phalman et al. 1984). Serum NSE concentration was found to be significantly increased in infants with asphyxia (Massaro et al. 2012). Increased serum and cerebrospinal fluid NSE concentrations have been found in newborn infants with hypoxic ischaemic encephalopathy (Celtik et al. 2004; Roka et al. 2012; Lv et al. 2015). Liu et al. (2005) reported that NSE levels were significantly increased in patients with hypoxic ischaemic encephalopathy with poor prognosis. Ok et al. (2022) reported a statistically significant decrease in NSE concentration in perinatal calves with hypoxic ischaemic encephalopathy compared to healthy calves. Ider et al. (2023) determined that NSE concentrations in hypoglycaemic diarrhoea calves were significantly higher than in healthy calves. In the present study, a statistically significant increase (*p* < 0.001) in serum NSE concentration was found in dogs with neurological distemper compared to control uninfected dogs (Table 4). Consistent with the report of many researchers that a significant increase in serum NSE concentration was observed in brain damage due to hypoxic ischaemic encephalopathy in infant (Celtik et al. 2004; Roka et al. 2012; Lv et al. 2015) and calves (Ider et al. 2023), a significant increase in serum NSE concentration was also found in dogs with neurological of distemper. High serum NSE concentrations in dogs with neurological distemper have been associated with the passage of NSE from the nerves into the bloodstream as a result of CDV damage to neurons (Leviton and Dammann 2002).

It has been reported that GFAP release increased due to the development of hypoglycaemia‐related brain damage in rats with experimentally induced hypoglycaemia (Afsari et al. 2008; Sahin et al. 2013). Significant increases in serum GFAP levels have been found in infants with neonatal encephalopathy (Yang et al. 2022) and hypoxic–ischaemic encephalopathy (Florio et al. 2010). They reported that there was no increase in serum GFAP concentration in perinatal calves with moderate and severe hypoxic ischaemic encephalopathy and the reason for the lack of increase in serum GFAP concentration may be due to the fact that this biomarker is a protein found in astrocytes (Ok et al. 2022). Unless astrocytes are severely damaged, their structures do not deteriorate and GFAP is not sufficiently released into the blood (Noorishakdam et al. 2020). Ennen et al. (2011) reported that there was no increase in GFAP level in structural brain damage in newborn babies. Ider et al. (2023) found that GFAP levels were significantly higher in hypoglycaemic calves than in healthy calves and suggested that this increase may be associated with glial damage, astrocyte activation (Kawata et al. 2016; Tomita et al. 2020) and a neuroprotective role (Afsari et al. 2008; Hashish et al. 2015). In the present study, a statistically significant increase (*p* < 0.001) in serum GFAP concentration was found in dogs with neurological distemper compared to control uninfected dogs (Table 4). The primary cause of elevated serum GFAP concentrations is the activation of astrocytes following brain injury and regional necrosis (Kawata et al. 2016). Astrocytes play a vital role in maintaining neuronal homeostasis in the brain by providing alternative energy to neurons under hypoglycaemic conditions (Bakken et al. 1998). In neurological distemper, damage occurs in both neurons and glial cells (astrocytes). As a result of damage to these cells, GFAP present in the cell passes into the bloodstream and causes blood GFAP levels to rise. In addition, blood GFAP levels rise due to increased astrocyte activation as a result of hypoglycaemia (Ider et al. 2023). We believe that high serum GFAP concentrations in dogs with neurological distemper may be related to its release from astrocytes to repair damaged neurons or its entry into the bloodstream when astrocytes are damaged.

ADM, a hypotensive vasodilator peptide, is synthesised in the organism as ADM preproadrenomodulin. The primary function of ADM, which has numerous biological effects, is to increase cyclic adenosine monophosphate production (Di Iorio et al. 2004). A significant increase in serum ADM concentration has been reported in patients with acute ischaemic stroke and acute intracranial haemorrhage (Somay et al. 2007; Chen et al. 2014; Whang et al. 2014; Zhang et al. 2014; Serrano‐Ponz et al. 2016). Conversely, Mitome‐Mishima et al. (2014) found that the serum concentration of ADM decreased in mice with ischaemic brain injury due to prolonged cerebral hypoperfusion. Similarly, Ok et al. (2022) observed a decrease in ADM concentration in perinatal calves with hypoxic–ischaemic encephalopathy, and Mahmoodazdeh et al. (2020) observed a decrease in ADM concentration in humans with hypoxic–ischaemic encephalopathy. These authors attributed the decrease to the breakdown of ADM by oxidative stress products produced intensely.Particularly in newborn babies, the products of oxidative stress can lead to high oxygen consumption and low antioxidant levels during the transition from the foetal to the neonatal period, inadequate ability of the brain to scavenge free radicals and increased susceptibility to free radicals, and damage to central nervous system tissues (Ferriero 2004; Buonocore and Groenendaal 2007; Ok et al. 2021). Ider et al. (2023) reported that serum ADM concentration did not increase in hypoglycaemic calves. In the present study, serum ADM concentration in dogs with neurological distemper was not statistically different (*p* > 0.05) compared to control uninfected dogs (Table 4). While the results of our study do not agree with those of many researcher, they are consistent with the findings of Ider et al. (2023). The probable reason for the lack of increase in serum ADM levels in this study is that this biomarker changes in acute brain damage. Since distemper is in its chronic phase, we believe that ADM may remain at normal levels in chronic brain damage.

ACTA is a protein that plays important biological effects in mesoderm induction, neuron cell differentiation, haematopoiesis, and reproductive physiology. ACTA has been shown to be released at high levels in preterm infants to prevent brain damage caused by cerebellar hypoxia (Mukerji et al. 2009). On the other hand, it has been reported that ACTA, together with other markers of brain damage in preterm infants, may be a useful marker for early detection of risk of neonatal brain damage (Metallinou et al. 2021; El‐Mazzahy et al. 2022). On the contrary, Ok et al. (2022) found a significant decrease in serum ACTA concentration in perinatal calves with hypoxic–ischaemic encephalopathy and attributed this decrease to the destruction of this protein by oxidative stress products, which are intense in hypoxic encephalopathy caused by asphyxia in calves. Similarly, Ider et al. (2023) found low ACTA concentrations in hypoglycaemic calves. It has been reported that ACTA concentration increases in the early stages of neonatal hypoxic–ischaemic encephalopathy (Lv et al. 2015). In the present study, the serum ACTA concentration of dogs with neurological distemper was not statistically different (*p* > 0.05) compared to control uninfected dogs (Table 4). The reason for the absence of an increase in serum ACTA concentration in dogs with neurological distemper may be related to the chronic phase of the disease. Indeed, contrary to the report by Lv et al. (2015) that serum ACTA concentrations increase in the early stages of neonatal hypoxic–ischaemic encephalopathy, we believe that ACTA levels may not increase in chronic phase brain damage.

CK‐BB is an isoenzyme found in neurons and astrocytes. Creatine kinase‐BB is released by astrocytes between glial cells (Tachikawa et al. 2004). CK‐BB is found at high levels in the brain and its activity increases in peripheral blood in brain injury (Lyons and Kettenmann 1998). Sweet et al. (1999) reported that blood CK‐BB enzyme activity increased in infants with perinatal asphyxia. On the contrary, Ok et al. (2022) found that serum CK‐BB enzyme activity was significantly decreased in perinatal calves with hypoxic–ischaemic encephalopathy, and the probable cause of this decrease was attributed to the destruction of this protein by oxidative stress products, which are intensively formed due to hypoxaemia caused by asphyxia in calves. Ider et al. (2023) found that CK‐BB concentration increased in hypoglycaemic calves and attributed this increase to the development of brain damage. On the other hand, it has been reported that serum CK‐BB enzyme activity increases for a short time and then decreases after brain injury (Mitome‐Mishima et al. 2014). The increase in CK‐BB enzyme activity in hypoglycaemia cases has been associated with the role of CK on brain energy demand and ATP production (brain energy haemostasis) (Tachikawa et al. 2004). In the present study, a statistically significant increase (*p* < 0.00) in serum CK‐BB concentration was found in dogs with neurological distemper compared to control uninfected dogs (Table 4). The results of our study were consistent with the findings of many researchers (Lyons and Kettenmann 1998; Sweet et al. 1999; Tachikawa et al. 2004; Ider et al. 2023), but inconsistent with the findings of many others (Mitome‐Mishima et al. 2014; Ok et al. 2022). Increased serum CK‐BB enzyme activity in dogs with neurological distemper has been associated with the intense passage of CK‐BB enzyme found in neurons and astrocytes into the bloodstream as a result of damage caused by CDV in neurons and astrocytes (Tachikawa et al. 2004).

Serum IgG levels were found to be significantly increased in both serum and CSF in distempered dogs with encephalitis (Jhonson et al. 1988). Many researchers (Calıskan and Burgu 2007; McRee et al. 2014) have determined serum IgM and IgG seropositivity in dogs with distemper. Saltık and Kale (2020) determined canine distemper virus‐specific IgM and IgG seropositivity in the acute phase and IgG seropositivity in the late phase of canine distemper. In the present study, serum IgG and IgM concentrations of dogs with neurological distemper were significantly elevated (*p* < 0.001) compared to control uninfected dogs (Table 4). Increased serum IgG levels in dogs with neurological distemper were consistent with the findings of many researchers (Jhonson et al. 1988; McRee et al. 2014; Saltık and Kale 2020; Sathe and Cusick 2023). This increase is related to the increased production of IgG as a humoral immune response in the late stages of the disease. On the other hand, many researchers (Von Messling et al. 1999; Cuesta et al. 2004; Alfona et al. 2022; Sathe and Cusick 2023)^,^ have reported that an increase in serum IgM levels may indicate that the animal has had a recent infection. The significant increase in serum and cerebrospinal fluid IgM levels in dogs with neurological distemper in the present study may be associated with the prolonged of IgM seropositivity in dogs with distemper (Waner et al. 2003). We think that IgM seropositivity in distemper dogs persists for a long time. Indeed, the report of Appel and Summers (1999) that seropositivity persists for 5 weeks to 3 months in dogs with distemper supports our conclusion.

The main limitation of our study is the limited number of animals, which may affect the significance of some biomarkers. The authors recommend that the current results be evaluated with a larger number of animals. Additionally, the fact that necropsies were not performed on all deceased dogs and that histopathological examination showing brain damage and immunohistochemistry analysis were not conducted can also be considered a limitation. These issues should be addressed in future studies.

In conclusion, it was demonstrated that the CDV virus causes significant damage to neurons and glial cells in canine neurological distemper, and that brain damage biomarkers can be clinically used to determine the extent of brain damage. It was determined that the biomarkers NSE, GFAP, and CK‐BB in dogs with neurological distemper have useful diagnostic significance in determining brain damage.

## Author Contributions

**MMK and MO**: designed and writing – original draft preparation, data analysis, conceptualisation, methodology and investigation. **OA**: performed biomarkers analysis and PCR analysis performed. All authors contributed to the article and approved the submitted version.

## Funding

This research was supported by the Selcuk University Scientific Research Project Office with project number 21212049.

## Ethics Statement

The study was conducted with the approval of the Selcuk University Veterinary Faculty Experimental Animal Production and Research Center Ethics Committee with decision number 2021/118.

## Conflicts of Interest

The authors declare no competing interests.

## Data Availability

The data that assist the results of the study are available from the corresponding author upon reasonable request.
